# Integrative system biology analyses of CRISPR-edited iPSC-derived neurons and human brains reveal deficiencies of presynaptic signaling in FTLD and PSP

**DOI:** 10.1038/s41398-018-0319-z

**Published:** 2018-12-13

**Authors:** Shan Jiang, Natalie Wen, Zeran Li, Umber Dube, Jorge Del Aguila, John Budde, Rita Martinez, Simon Hsu, Maria V. Fernandez, Nigel J. Cairns, Dominantly Inherited Alzheimer Network (DIAN), International FTD-Genomics Consortium (IFGC), Oscar Harari, Carlos Cruchaga, Celeste M. Karch

**Affiliations:** 1 Department of Psychiatry, Washington University School of Medicine, 660S. Euclid Ave. Campus Box 8134, St. Louis, MO 63110 USA; 2 Hope Center for Neurological Disorders, Washington University School of Medicine, 660S. Euclid Ave. Campus Box 8111, St. Louis, MO 63110 USA; 3 Department of Pathology and Immunology, Washington University in St. Louis, School of Medicine, 660S. Euclid Ave, Campus Box 8118, Saint Louis, MO 63110 USA

## Abstract

Mutations in the microtubule-associated protein tau (*MAPT*) gene cause autosomal dominant frontotemporal lobar degeneration with tau inclusions (FTLD-tau). *MAPT* p.R406W carriers present clinically with progressive memory loss and neuropathologically with neuronal and glial tauopathy. However, the pathogenic events triggered by the expression of the mutant tau protein remain poorly understood. To identify the genes and pathways that are dysregulated in FTLD-tau, we performed transcriptomic analyses in induced pluripotent stem cell (iPSC)–derived neurons carrying *MAPT* p.R406W and CRISPR/Cas9-corrected isogenic controls. We found that the expression of the *MAPT* p.R406W mutation was sufficient to create a significantly different transcriptomic profile compared with that of the isogeneic controls and to cause the differential expression of 328 genes. Sixty-one of these genes were also differentially expressed in the same direction between *MAPT* p.R406W carriers and pathology-free human control brains. We found that genes differentially expressed in the stem cell models and human brains were enriched for pathways involving gamma-aminobutyric acid (GABA) receptors and pre-synaptic function. The expression of GABA receptor genes, including *GABRB2* and *GABRG2*, were consistently reduced in iPSC-derived neurons and brains from *MAPT* p.R406W carriers. Interestingly, we found that GABA receptor genes, including *GABRB2* and *GABRG2*, are significantly lower in symptomatic mouse models of tauopathy, as well as in brains with progressive supranuclear palsy. Genome wide association analyses reveal that common variants within *GABRB2* are associated with increased risk for frontotemporal dementia (*P* < 1 × 10^−3^). Thus, our systems biology approach, which leverages molecular data from stem cells, animal models, and human brain tissue can reveal novel disease mechanisms. Here, we demonstrate that *MAPT* p.R406W is sufficient to induce changes in GABA-mediated signaling and synaptic function, which may contribute to the pathogenesis of FTLD-tau and other primary tauopathies.

## Introduction

Frontotemporal lobar degeneration (FTLD) encompasses several disease entities that are distinguished by the molecular pathology of neuronal and glial inclusion bodies. FTLD with tau-immunoreactive inclusion bodies (FTLD-tau) make up about 50% of cases. These primary tauopathies include progressive supranuclear palsy (PSP), corticobasal degeneration (CBD), and Pick disease^1,2^. Macroscopically, FTLD-tau is characterized by frontal and temporal lobar atrophy in contrast to Alzheimer disease (AD) where atrophy is more generalized. The insular cortex and basal ganglia are frequently affected. Microscopically, FTLD-tau, as with all FTLD entities, displays neuronal loss and gliosis in affected areas. FTLD-tau is clinically heterogeneous and may present with a broad spectrum of phenotypes including behavioral, language, memory and motor disturbances^3^. In a subset of FTLD-tau cases, rare mutations in the microtubule associated protein tau (*MAPT)* gene are sufficient to cause disease^4,5^. More than 50 mutations in *MAPT* have been reported to cause FTLD-tau and are located primarily in exons 9–13^6^. Despite the clear association of *MAPT* mutations with FTLD-tau, we have little understanding of the downstream events that lead to neurodegeneration and dementia.

The mechanism(s) by which *MAPT* mutations disrupt tau metabolism and contribute to disease are poorly understood in part due to the complexities of the *MAPT* gene. The *MAPT* gene in the central nervous system is alternatively spliced to produce 6 isoforms that differ based on the presence of the N-terminal insert (0N, 1N, 2N) and the number of microtubule binding repeats (MTBR; 3R, 4R). Several mechanisms have been proposed to explain how *MAPT* mutations cause disease: abnormal *MAPT* splicing, altered microtubule binding kinetics, impaired degradation, tau accumulation and aggregation, among others^7^.

*MAPT* p.R406W heterozygous carriers are notable in presenting more commonly with AD-like progressive memory decline and a protracted clinical course that may last decades^8–10^. Neurofibrillary tangles of *MAPT* p.R406W contain 3R and 4R tau and are virtually indistinguishable from those of AD; they are present largely in the frontal and temporal neocortices and hippocampus^11^. In a transgenic mouse model, expression of human *MAPT* p.R406W leads to age-dependent accumulation of tau aggregates, reduction of tau in axonal compartments and neurodegeneration^12,13^. *MAPT* p.R406W does not alter tau-mediated microtubule dynamics nor does it induce filament formation in vitro^14–16^. *MAPT* p.R406W may block the interaction of tau with neuronal membranes and impede neurite outgrowth^17,18^. However, the pathogenic events triggered by the expression of the mutant tau protein remain poorly understood.

Human induced pluripotent stem cell (iPSC)-derived neurons have emerged as a powerful cellular system to model the complexities of pathological gene expression, particularly in the early stage of disease, in the context of a non-neoplastic human genome. When coupled with genome editing by CRISPR/Cas9, this system allows for molecular events to be pinpointed specifically and to impute causality for different molecular phenotypes. However, in the context of neurodegenerative diseases, such as FTLD-tau, it remains unclear whether the stem cell models capture events that are critical and proximal to disease pathogenesis. Here, we propose a novel integrative approach to identify molecular drivers of neurodegenerative disease using a bioinformatic pipeline that reduces the risk of false-positives arising from small sample sizes and improves our ability to identify disease-relevant pathways.

To begin to understand the molecular events caused by the *MAPT* p.R406W mutation, we sought to leverage stem cell models and human brain tissue to identify molecular changes that are triggered by *MAPT* p.R406W and that lead to human disease. We found that genes differentially expressed in the stem cell models and brains were enriched for pathways involving gamma-aminobutyric acid (GABA) receptors and pre-synaptic function. The expression of GABA receptor genes, including *GABRB2* and *GABRG2*, were consistently reduced in iPSC-derived neurons and brains from *MAPT* p.R406W carriers, PSP brains, and mouse models of tauopathy. Here, we demonstrate that *MAPT* p.R406W is sufficient to induce changes in GABA-mediated signaling and synaptic function, which contribute to the pathogenesis of FTLD-tau.

## Materials and methods

### Patient consent

The Washington University School of Medicine Institutional Review Board reviewed the protocol of the Knight Alzheimer Disease Research Center (ADRC) Neuropathology Core, from which clinically and neuropathologically well-characterized brain tissues were obtained. As tissue was obtained postmortem it was exempt from IRB approval. Research participants provided autopsy consent limited to removal of the brain. All data were analyzed anonymously.

Skin punches were performed following written informed consent from the donor. The informed consent was approved by the Washington University School of Medicine Institutional Review Board and Ethics Committee (IRB 201104178 and 201306108). The consent allowed for use of tissue by all parties, commercial and academic, for the purposes of research but not for use in human therapy.

### iPSC generation and genome engineering

Dermal fibroblasts from a *MAPT* p.R406W carrier (F11362) were transduced with non-integrating Sendai virus carrying OCT3/4, SOX2, KLF4, and cMYC (Life Technologies)^19,20^. Cells that showed morphological evidence of reprogramming were selected manually. iPSC lines were characterized using standard methods^19^. Each resulting clone was analyzed for pluripotency markers by immunocytochemistry (ICC) and quantitative PCR (qPCR); for spontaneous differentiation into the three germ layers by ICC and qPCR; and for chromosomal abnormalities by karyotyping (see below for details). Mutation status was confirmed in newly generated iPSC by Sanger sequencing (Supplemental Fig. 1). Cell lines were confirmed to be free of mycoplasma.

iPSC that were heterozygous for *MAPT* p.R406W were edited using CRISPR/Cas9 as previously reported^21^. Briefly, allele-specific guideRNAs (gRNAs) were designed for the mutant allele. gRNAs were designed to have at least 3 bp of mismatch to any other gene in the human genome and validated for activity using the T7E1 assay. In this assay, the T7E1 enzyme recognizes and cleaves non-perfectly matched DNA. To prepare cells for editing, iPSC colonies were dissociated into single cells via incubation in Accutase for 10 min. Single cell iPSC cultures were maintained on Matrigel supplemented with fibronectin and cultured in mTesR. To edit cells, human iPSCs were nucleofected with 1 μg pMaxGFP (used to assess nucleofection efficiency), 1 μg gRNA, 3 μg Cas9, and 300 μM single stranded oligodeoxynucleotides (ssODN) and the P3 Primary Cell 4D reaction mix (Lonza). At least 96 clones were screened for editing. We routinely selected 2–5 edited clones for expansion and characterization. In addition to edited clones, we selected 1–2 unedited clones that were exposed to the genome-editing pipeline but remained unmodified. Characterization of edited and unedited clones included qPCR and ICC for pluripotency markers, karyotyping and Sanger sequencing of on and predicted off-target sites (Supplemental Fig. 1).

### Immunocytochemistry

Immunocytochemistry was performed to confirm pluripotency of the cultures (Supplemental Fig. 1). Culture media was aspirated, and cells were washed and fixed with 4% paraformaldehyde (Sigma). Cells were washed and permeabilized with permeabilization buffer (0.1% Triton X-100 in PBS). Cells were then blocked in 0.1% bovine serum albumin (BSA; Sigma) and treated with primary and secondary antibodies diluted in 0.1% BSA. Immunostained cells were then imaged (Nikon Eclipse 80i fluorescent microscope). The following antibodies were used: SOX2, Tra1-60 (Abcam, San Francisco, CA, USA); DAPI (Life Technologies); Alexa488 anti-rabbit (Life Technologies); and Alexa594 anti-mouse (Life Technologies).

### Quantitative PCR

To determine whether iPSC clones express endogenous markers of pluripotency, we performed qPCR (Supplemental Fig. 1). RNA was extracted from cell pellets with an RNeasy kit (Qiagen), following the manufacturer’s protocol. Extracted RNA (10ug) was converted to cDNA by PCR using the High-Capacity cDNA Reverse Transcriptase kit (Life Technologies). Gene expression was measured in iPSCs using qPCR as previously described (*SOX2*, *POU5F1*, *LIN28A*, *NANOG*, *B3GALT5*, *PODXL*, *SEV*)^22^. Primers specific to *GAPDH* were used as a loading control.

### Karyotyping

Chromosomal abnormalities were assessed by G-band karyotyping after clonal isolation of iPSC and after genome editing (Supplemental Fig. 1).

### Differentiation of iPSC into cortical neurons

One *MAPT* p.R406W iPSC donor and one isogenic control iPSC line were selected for evaluation by RNA-seq. To generate neurons, iPSCs were harvested for neural aggregate formation upon reaching 75–85% confluency. iPSCs were dissociated with Accutase (MP Biomedicals) and pelleted by centrifugation. Cells were counted and resuspended in mTesR1 supplemented with the ROCK inhibitor, Y-27632 (10 µM), to achieve 450,000–650,000 cells/mL. Then, 100 µL/well of iPSC suspension was plated into a v-bottom 96-well plate. The v-bottom plate was centrifuged at 750 rpm for 3 min to pellet the iPSCs and promote formation of spheres. Cells were then incubated at 37 °C, 5% CO_2_ for 24 h. Neurospheres were cultured for 5 additional days with daily media change in Neural Induction Medium (NIM; Stemcell Technologies).

Per manufacturer’s guidelines, on Day 5 of neural aggregate formation, spheres were washed with 100 µL of DMEM/F12. Then, 100 µL of NIM was added to each well. Spheres were transferred from the conical wells into a 6 well plate, pre-coated with poly-Ornithin (PLO)/Laminin (Millpore-Sigma), at a density of 32 spheres per well. Daily media changes followed with NIM. Spheres were monitored daily for formation of neural rosette structures. Neural rosettes were harvested when spheres had completely flattened and clusters were clearly visible (3–7 days after plating; line dependent). Neural rosettes were harvested by aspirating spent medium, washing with DMEM/F12, and then adding 1 mL of Neural Rosette Selection reagent (Stemcell Technologies) to each well for 1 h at 37 °C. Rosette clusters were then detached by pipetting with DMEM/F12. Rosette clusters were transferred into a conical tube, centrifuged at 750 rpm for 3 min, and then either cryopreserved or plated for neural progenitor cell expansion. NPCs were cultured on PLO and Laminin-coated plates and terminal differentiation was initiated with the addition of cortical maturation media (Neurobasal Media (Life Technologies) supplemented with B27 (GIBCO), BDNF (Peprotech), GDNF (Peprotech), cAMP (Sigma), and L-glutamate (Sigma)). Neurons were maintained for 6 weeks.

### Human brain tissue

Clinically and neuropathologically well-characterized brain tissue was obtained from the Knight ADRC and the Dominantly Inherited Alzheimer Network (DIAN), both at Washington University in St Louis. Genotyping was performed to determine whether brain tissue carried a pathogenic mutation as described previously^23^. Brain tissue from the insular cortex was obtained from *MAPT* p.R406W carriers (*n* = 2) and pathology-free control cases (*n* = 2). We also used brain tissue from the parietal cortex from sporadic FTLD-TDP (*n* = 11), sporadic late onset AD (LOAD; *n* = 80), *PSEN1* (p.A79V, p.N135S, p.I143T, p.H163R, p.S170F, p.G206A, p.G217R, p.L226R, p.I229F, p.S290C, p.T245P, p.C410Y, p.A431E) with AD (*n* = 18), and pathology-free control cases (*n* = 11). In one case, we evaluated parietal and insular cortices from the same case (control no. 10 and 13, respectively; Supplementary Table 1). In general, control brains were matched based on age, sex and DV200, defined as the percentage of RNA fragments >200 nucleotides in size. Specifically, *MAPT* p.R406W and control brains were matched based on sex and DV200. ANNOVA was performed to demonstrate that there was no statistically significant difference between covariates (*P*-value = 0.988 for sex and *P*-value = 0.659 for DV200).

To evaluate whether genes differentially expressed in iPSC-derived neurons and brains from *MAPT* p.R406W compared with controls were also differentially expressed in primary tauopathies, we examined previously generated gene expression profiles from the temporal cortex and cerebellum of 80 control and 84 progressive supranuclear palsy (PSP) brains (syn5550404)^24^. Brain samples used in this study are summarized in Table 1 and Supplemental Table 1.

**Table 1:** Characteristics of brain samples analyzed in this study

Sample group	Neuropathologic diagnosis	Sample size	Brain region	Age (years)^a^	Female (%)	DV200^a^	Reference
*MAPT* p.R406W	FTLD-Tau	2	Insular cortex	69.46 ± 3.88	50%	85.33 ± 11.55	^23^
Controls	None	2	Insular cortex	71.81 ± 2.14	50%	88.5 ± 2.12	^23^
Sporadic FTLD	FTLD-TDP	11	Parietal cortex	74.09 ± 17.00	45.5%	89.45 ± 4.16	^23^
Late-onset AD	AD	80	Parietal cortex	85.83 ± 6.88	61.25%	89.39 ± 3.81	^23^
ADAD^b^	AD	18	Parietal cortex	50.17 ± 12.03	37.5%	86.06 ± 4.22	^23^
Controls	None	11	Parietal cortex	85.01 ± 9.06	45.5%	90.09 ± 1.87	^23^
PSP	PSP	84	Temporal cortex	73.96 ± 6.54	39.29%	NA	^24^
PSP	PSP	84	Cerebellum	73.95 ± 6.54	39.29%	NA	^24^
Controls	None	80	Temporal cortex	82.55 ± 8.80	48.75%	NA	^24^
Controls	None	79	Cerebellum	82.67 ± 8.11	49.37%	NA	^24^

^a^Mean ± standard deviation

^b^*PSEN1* A79V, N135S, I143T, H163R, S170F, G206A, G217R, L226R, I229F, S290C, T245P, C410Y, A431E

*NA* not available

### RNA extraction, library construction, and sequencing

RNA was extracted from frozen brain tissues and iPSC-derived neuronal cell pellets using Tissue Lyser LT and RNeasy Mini Kit (Qiagen, Hilden, Germany) following the manufacturer’s instructions. RNA yields of individual samples were quantified by the Quant-iT RNA Assay (Life Technologies) on the Qubit Fluorometer (Fisher Scientific). Quality of yielded RNA of individual samples was assessed by the DV200 metric. DV200 of individual samples was measured with the RNA 6000 Pico Assay using the Bioanalyzer 2100 (Agilent Technologies). cDNA libraries of individual samples were constructed using the TruSeq Stranded Total RNA Sample Prep with Ribo-Zero Gold kit (Illumina) and then sequenced by the HiSeq 4000 (Illumina) at the McDonnell Genome Institute at Washington University in St. Louis with a mean of 58.14 ± 8.62 million reads as previously described^23^.

### Quality control, alignment and quantification

Quality control, alignment and quantification was performed on all newly generated RNA-seq samples, as well as publically available sequencing reads from PSP and control brains (syn5550404)^23,24^. After converting the sequencing reads to FASTQ format, FastQC was used to check quality of the RNA-seq data. STAR (v. 2.5.2b) was then used to align the FASTQ files to human GRCh37 primary assembly^25^. We used the primary assembly and aligned reads to the assembled chromosomes, unlocalized and unplaced scaffolds, and discarded alternative haploid sequences. Sequencing metrics, including coverage, distribution of reads in the genome, ribosomal and mitochondrial contents, and alignment quality, were further obtained by applying Picard CollectRna-SeqMetrics (ver 2.8.2) to detect sample deviation.

Aligned and sorted bam files were loaded into IGV to visually identify target variants^26^. Samples carrying unexpected or missing expected variants were labeled as potentially swapped samples. In addition, variants were called from RNA-seq following BWA/GATK pipeline^27,28^. The identities of the samples were later verified by performing identity-by-descent analysis against genomic typing from genome-wide association study chipsets.

After alignment, Salmon (v. 0.7.2) was used to quantify expression levels of individual genes included in the GENCODE reference genome (GRCh37.75)^29^. We applied quality control, alignment and quantification as previously described^23^.

### Differential expression analysis

We tested for differential expression using the R (v.3.4.2) package DESeq2 (v.1.18.1)^30^, that uses the negative binomial distribution to calculate the statistical significance. DESeq2 reports *P* values and False Discovery Rate (FDR)—corrected *P* values for individual genes—determined using a Wald test. It has previously been demonstrated that there are many fewer spurious differentially expressed genes (DEGs) for a given false discovery rate (FDR) when isogenic controls are used with paired mutant lines rather than populations controls, even with smaller sample sizes^31^. A paired analysis (mutant vs. isogenic controls) is a highly sensitive and specific approach provided a more stringent FDR threshold is adopted^31^. Thus, we applied the more stringent Benjamini-Yekutieli FDR (B-Y FDR) correction to guarantee the specificity of the differentially expressed genes^32^. B-Y extends Benjamini -Hochberg (B-H) by modeling and penalizing for positive dependency, such as the comparison of multiple treatments with a single control, correlated tests or effects of latent variables on endpoints of same brain. Genes with B-Y FDR-corrected *P* values less than 0.05 were considered differentially expressed. Volcano plots were generated using built-in R function plot. Heat maps of differentially expressed genes were generated using R package pheatmap (v.1.0.8) (https://cran.r-project.org/web/packages/pheatmap/index.html).

### Digital deconvolution of bulk RNA-seq

To determine whether the cellular composition of iPSC-derived neurons from *MAPT* p.R406W and isogenic controls were similar, we applied a machine learning approach to digitally deconvolve bulk RNA-seq data to reveal information as to the percentage of neurons, astrocytes, microglia and oligodendrocytes in the cultures^23,33^. To infer cellular composition of the iPSC-neurons, we assembled and validated a reference panel composed of cell type specific samples and genes. Then we applied a digital deconvolution algorithm termed semi-supervised non-negative matrix factorization algorithm (ssNMF^34^) to infer cellular composition structure.

### Functional annotation of differentially expressed genes

Functional annotation was performed on genes that were significantly differentially expressed in the same direction in both iPSC-derived neurons and brains from *MAPT* p.R406W carriers and controls. Gene relationships, including physical interaction, co-localization, pathway, shared protein domain, and genetic interaction, with or without co-expression among these verified differentially expressed genes were annotated using the Cytoscape plugin GeneMANIA (v. 3.4.1)^35^. Gene co-expression data were derived from brain tissues of donors free of neuropathology^36^. Gene topological properties were calculated using Cytoscape built-in tool NetworkAnalyzer. To identify enrichments in gene ontologic features, biological pathways and drug targets, we used Webgestalt (http://www.webgestalt.org/)^36^. In Webgestalt, the 61 differentially expressed genes were input as a target gene set and all sequenced genes were input as the reference gene set. Enrichments with B-H FDR-corrected *P* values less than 0.05 were considered significantly enriched.

### Mouseac

To determine whether genes differentially expressed in iPSC-derived neurons and brains from *MAPT* p.R406W carriers and controls were similarly altered in a mouse model of tauopathy, we evaluated gene expression in the *MAPT* p.P301L transgenic mouse model (www.mouseac.org)^37^. We focused on the GABA receptor genes that were differentially expressed in iPSC-derived neurons, FTLD-tau brains and PSP brains compared with controls. Briefly, microarray gene expression data was evaluated from two brain regions (cortex and hippocampus) from non-transgenic C57Bl/6 J (2, 4, 8, 18 months; *n* = 4 per time point) and *MAPT* p.P301L transgenic mice under the control of a CaMKII promoter (2 months, *n* = 11, 4 months, *n* = 12; 8 months, *n* = 10; 18 months, *n* = 7). Gene expression levels were log2 transformed and expressed as a function of age. The normality of distribution was assessed using the Kolmogorov-Smirnov test. Between-group differences of normally distributed data were assessed using an unpaired *t*-test. The presence of neurofibrillary tangle (NFT) pathology was evaluated and scored as previously reported by immunohistochemistry^37^.

### Developmental expression of differentially expressed genes

To determine whether genes differentially expressed in iPSC-derived neurons from *MAPT* p.R406W carriers and isogenic controls (328 genes) change during development, we evaluated human brain expression data from BrainSpan (www.brainspan.org) across 11 developmental stages to generate a heat map^38^. Samples were grouped into developmental stages: embryonic (age 4–7 post-conception weeks (pcw)); early prenatal A (age 8–9 pcw); early prenatal B (age 10–12 pcw); early mid-prenatal A (age 13–15 pcw); early mid-prenatal B (age 16–18 pcw); late mid-prenatal (age 19–24 pcw); late prenatal (age 25–38 pcw); early infancy (age birth-5 months); late infancy (age 6–18 months); early childhood (age 19 months-5 years); late childhood (age 6–11 years); adolescence (age 12–19 years); and adulthood (20–40 years). Samples from each developmental stage were compared to samples from adulthood stage, which were used as the reference group to assess the developmental expression difference of differentially expressed genes. Sex and brain region were included as covariates in the analysis.

### Gene enrichment in FTD GWAS loci

To determine whether genes differentially expressed in iPSC-derived neurons and brains from *MAPT* p.R406W carriers and controls occur within loci that increase frontotemporal dementia (FTD) risk, we used summary statistics from a genome wide association study (GWAS) of FTD^39^. The International FTD-Genomics Consortium (IFGC) provided phase 1 FTD-GWAS summary statistic data, which consisted of 2154 FTD cases and 4308 controls with genotypes or imputed data at 6,026,384 SNPs. The FTD dataset included multiple clinical subtypes within the FTD spectrum: behavioral variant FTD, semantic dementia, progressive non-fluent aphasia and FTD with motor neuron disease^39^. The criteria for annotating an FTD-GWAS SNP to a given differentially expressed gene was that the GWAS SNP was located within 5 kb of the gene boundaries.

To determine whether the differentially expressed genes in *MAPT* p.R406W carriers were significantly enriched for FTD risk, improved gene-set enrichment analysis for GWAS (*i*-GSEA4GWAS) was employed^40,41^. The maximum −log(*P*-value) of the SNPs located within 5 kb of a gene boundary was assigned to represent the gene. Instead of the commonly used phenotype label permutation, SNP label permutations were implemented to generate the distribution of the enrichment score (*ES*). A gene set significance proportion based enrichment score (*SPES*) was calculated based on gene rank using the following equation: *SPES* = *k/K* × *ES*, where *k* is the proportion of significant genes of the gene set and *K* is the proportion of significant genes of the total number of genes in the GWAS. Gene-length bias was corrected by applying adaptive permutation in PLINK before *i*-GSEA4GWAS. Then, a comparative quantile-quantile (QQ) plot was used to demonstrate the differential association with FTD between all genes and the genes differentially expressed in *MAPT* p.R406W carriers.

To demonstrate the specificity of the enrichment of genes differentially expressed in iPSC-derived neurons and brains from *MAPT* p.R406W carriers compared to controls, *i*-GSEA4GWAS was also employed on genes differentially expressed in AD, schizophrenia (SCZ), bipolar disorder (BD) and autism spectrum disorder (ASD) for FTD risk. Genes differentially expressed in SCZ, BD, and ASD were from obtained from a recently published study^42^.

## Results

### Systems biology approach to identify molecular drivers of FTLD-tau

**Fig. 1:**
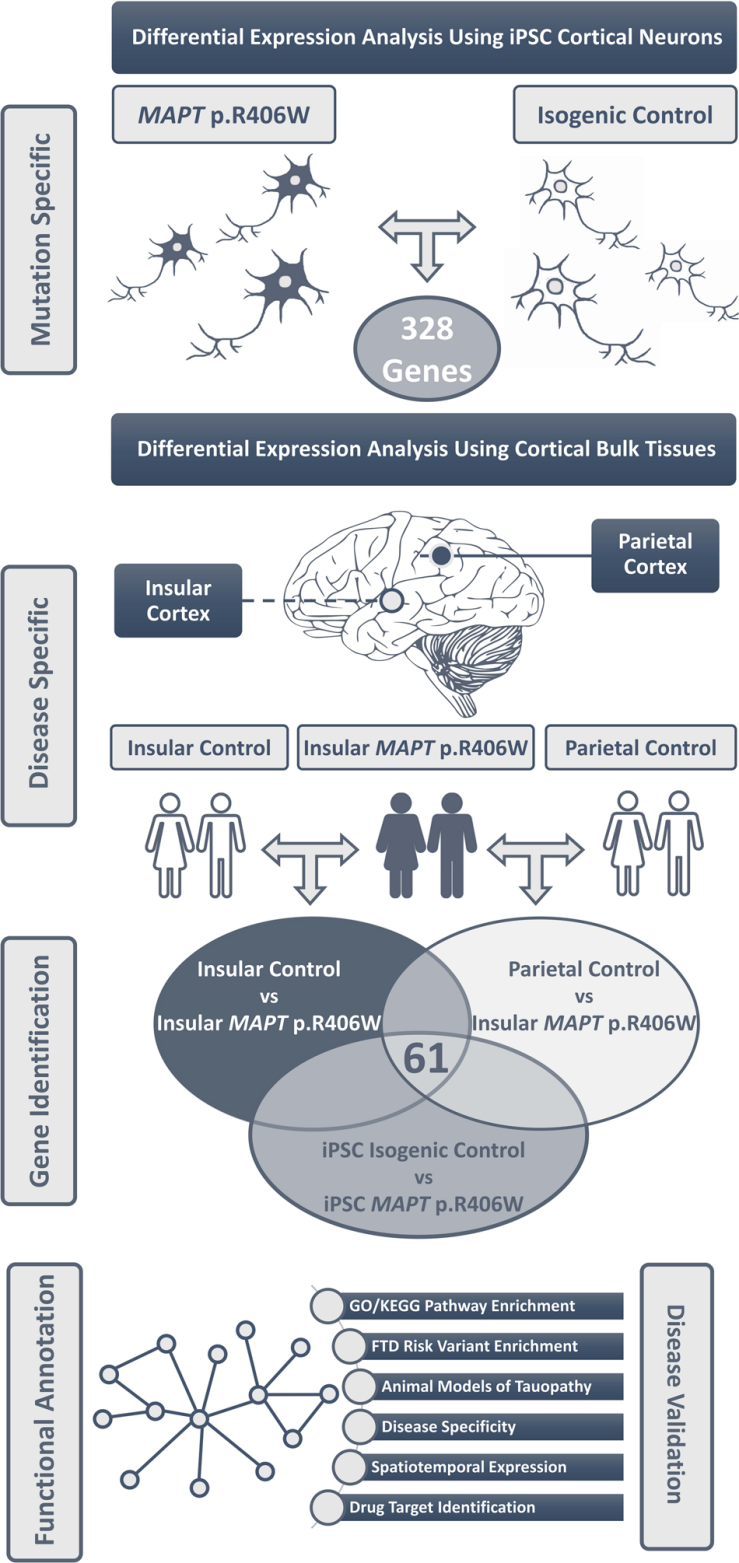
Integrative analysis to define genes and pathways dysregulated in FTLD with *MAPT* p.R406W. Human iPSC-derived cortical neurons from *MAPT* p.R406W carriers and isogenic controls served as a discovery cohort to identify genes affected by the *MAPT* p.R406W mutation (*n* = 328). To define the genes that are differentially expressed in disease, we sought to replicate the differential expression analysis in human brains from *MAPT* p.R406W carriers and non-carrier controls. We identified 61 genes that were differentially expressed in both paradigms (FDR B-Y < 0.05). These 61 genes were then functionally annotated and interrogated animal models of FTLD-tau

The goal of our study was to identify genes and networks that are critical drivers of disease pathogenesis in FTLD-tau downstream of *MAPT* mutations. As human brain tissue from FTLD with *MAPT* p.R406W mutation is rare, identifying and replicating genetic and molecular networks involved in the etiology of disease has been challenging, resulting in little focus outside of the tau protein. Thus, we sought to develop a novel integrative approach in which we coupled iPSC-derived neurons from *MAPT* p.R406W carriers and CRISPR/Cas9-generated isogenic controls as a discovery cohort with validation using human brain tissue to identify consistently differential expressed genes (Fig. 1). We also compared the differentially expressed genes in the iPSC-derived neurons vs. isogenic cell lines with those genes differentially expressed in autosomal dominant AD (*PSEN1* mutation carriers), FTLD-TDP, and PSP in order to determine if those genes were related to *MAPT* p.R406W, tau pathology or neurodegeneration in general. Finally, we performed pathway analysis and functional annotation to identify the main networks that lead to disease in *MAPT* p.R406W carriers and to identify novel potential therapeutically targets. Together, this approach led us to conclude that GABA-related molecular pathways are involved in FTLD with *MAPT* p.R406W mutation and PSP and may constitute novel targets in these diseases.

### *MAPT* p.R406W is sufficient to induce transcriptome-wide changes in iPSC-derived neurons

**Fig. 2:**
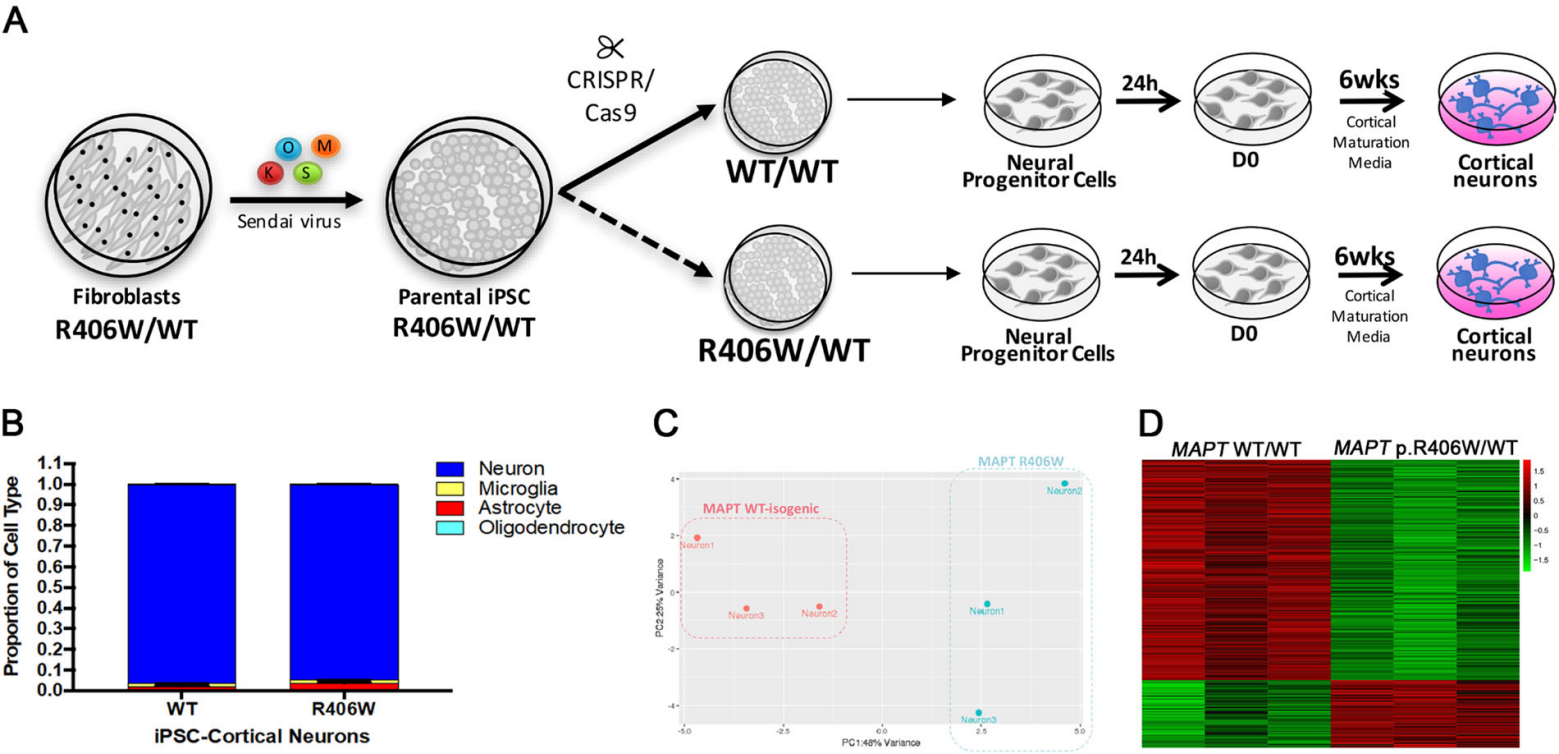
*MAPT* p.R406W is sufficient to induce transcriptome-wide changes in iPSC-derived cortical neurons. **a** Fibroblasts from a symptomatic *MAPT* p.R406W carrier were reprogrammed into iPSC. CRISPR/Cas9 was used to correct the mutant allele and to establish an isogenic control line. iPSCs from *MAPT* p.R406W and isogenic controls were differentiated into cortical neurons and cultured for 6 weeks prior to analysis. **b** Digital deconvolution was applied to bulk RNA-seq data to define the relative percentage of neurons, astrocytes, microglia and oligodendrocytes. We demonstrate that *MAPT* p.R406W and isogenic control cultures are similarly enriched in neurons (>98%). Graph represents mean ± SEM. **c** Principal Component Analysis demonstrates that *MAPT* p.R406W is sufficient to induce transcriptome-wide differences in gene expression (PC1: 48% variance). **d** Differential expression analysis reveals that 328 genes differ between iPSC-derived cortical neurons carrying *MAPT* p.R406W vs. isogenic control (FDR B-Y < 0.05). Gene expression represented as in a heat map. RNA-seq was analyzed in 3 biological replicates

To determine whether *MAPT* p.R406W is sufficient to induce molecular changes in human neurons that may lead to neuronal dysfunction, we leveraged human somatic cells isolated from *MAPT* p.R406W carriers^8^. Fibroblasts were isolated from a skin punch biopsy and reprogrammed into pluripotent cells using non-integrating Sendai virus expressing *KLF4*, *OCT4*, *SOX2*, and *cMYC* (Fig. 2a; Supplemental Fig. 1). Clonal lines were picked and expanded. Only those clones expressing endogenous pluripotency markers by immunohistochemistry and qPCR and that also displayed a normal karyotype were used in this study (Supplemental Fig. 1). Genetic background of individual donors is a large contributor to phenotypic variability in iPSCs;^43^ thus, cell lines from non-mutation carriers are not ideal controls. To define phenotypes driven specifically by the mutant allele, we used CRISPR/Cas9 genome editing to establish isogenic controls for donor iPSC line (Fig. 2a). All cell lines used in this study were fully characterized for pluripotency and chromosomal stability (Supplemental Fig. 1). One clone from the *MAPT* p.R406W and the isogenic, corrected control (*MAPT* WT) were then differentiated into cortical neurons using a growth factor-based approach (see Methods). After 6 weeks in cortical maturation medium, we collected RNA and evaluated the whole transcriptome by RNA-seq. At 6 weeks in culture, iPSC-derived neurons produce spontaneous action potentials, form functional synapses, and display a profile of tau isoform expression that is similar to tau found in the central nervous system^44^.

To begin to evaluate the impact of *MAPT* p.R406W on the overall transcriptome profile, we first sought to determine whether the *MAPT* p.R406W and isogenic controls exhibited a similar capacity to form neurons. We applied a digital deconvolution approach to predict the proportion of neurons, astrocytes, microglia and oligodendrocytes in the cultures^23^. We found that *MAPT* p.R406W and isogenic controls were similarly enriched for neuronal-specific genes (Fig. 2b). Additionally, we examined the expression of individual genes specific to cortical layer markers to determine whether the *MAPT* p.R406W mutation influenced survival of neuronal subtypes in the culture. Deep cortical layer markers (*TBR1*, *PCP4*, *TLE4*); upper cortical layer markers (*TLE1*, *SATB2*, *CUX1*, *POU3F2*); layer 4 marker (*RORB*); layer 5 marker (*BCL11B*); and subplate markers (*CTGF*, *NR4A2*)^45^ were not significantly differentially expressed between the *MAPT* p.R406W neurons and isogenic controls (Supplemental Table 2). Thus, we would predict that any transcriptome-wide changes between the *MAPT* p.R406W and isogenic controls are driven by molecular dysregulation within neurons rather than being a reflection of differences in cellular composition between cultures.

To determine whether the *MAPT* p.R406W mutation is sufficient to induce changes in gene expression in human neurons, we performed differential expression analyses. We found that the expression of the *MAPT* p.R406W mutation leads to a unique transcriptome profile that is significantly different compared to that of the isogeneic controls. Principal component analysis revealed that *MAPT* mutation explains 48% of the transcriptome-wide variance between *MAPT* p.R406W and isogenic controls (Fig. 2c), and the *MAPT* p.R406W was sufficient to discriminate the two groups: the mutation carriers from the isogeneic controls. These results indicate that the *MAPT* p.R406W is sufficient to shift the normal transcriptome state of human neurons and some of these changes could be involved in disease pathogenesis.

**Fig. 3:**
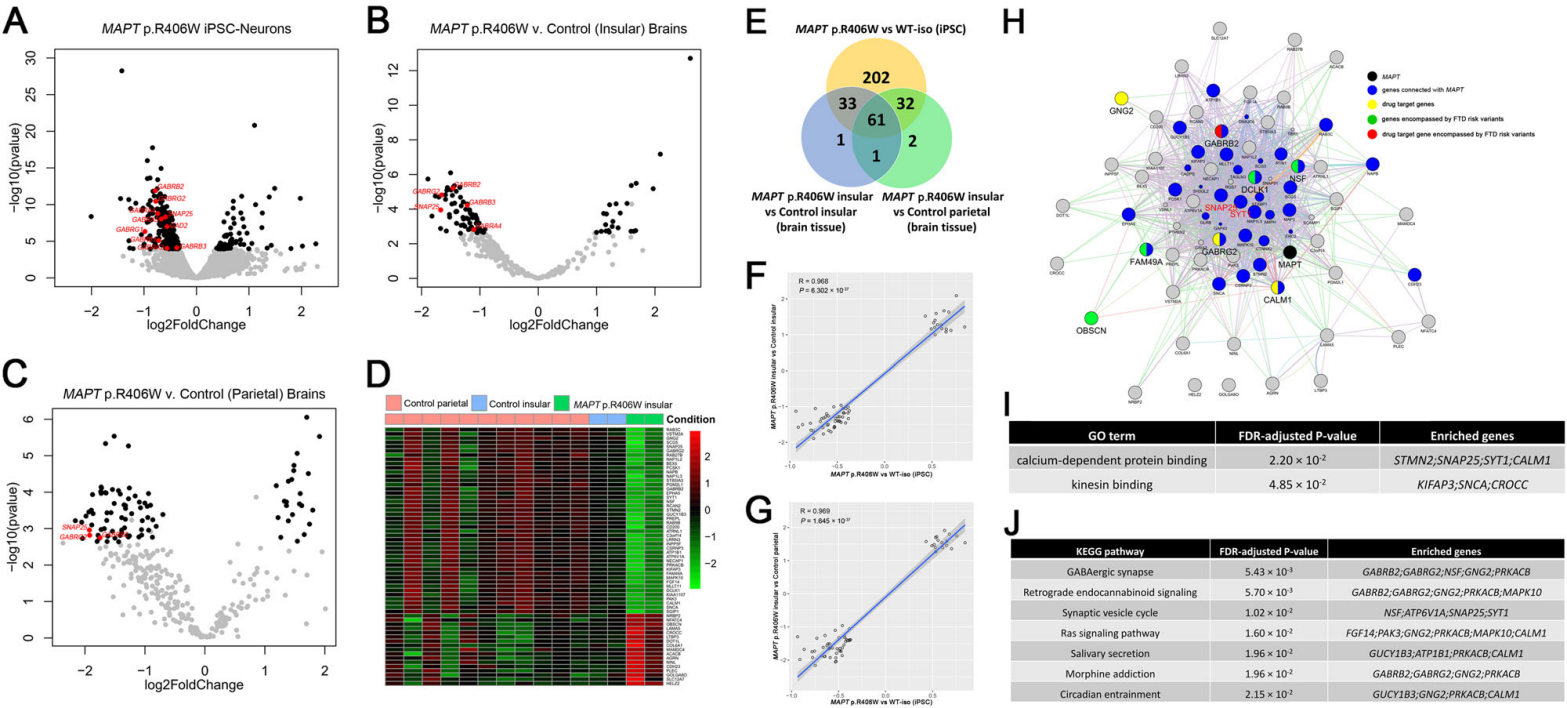
Replication and disease validation of genes differentially expressed in *MAPT* p.R406W brains. To determine whether the 328 differentially expressed genes in iPSC-derived neuronal models capture changes in gene expression that are relevant to human disease, we tested whether these 328 genes were differentially expressed in human brains from *MAPT* p.R406W carriers compared with normal controls. **a** Volcano plot showing log2 fold change between iPSC-derived cortical neurons carrying *MAPT* p.R406W vs. isogenic controls and the –log_10_ *P*-value for each gene. Black nodes: the 328 genes with FDR B-Y < 0.05. **b** Volcano plot of the 328 genes showing log2 fold change between *MAPT* p.R406W (insular cortex) and control (insular cortex) brains and the –log_10_ *P*-value for each gene. Black nodes: genes with FDR B-Y < 0.05. **c**. Volcano plot of the 328 genes showing log2 fold change between *MAPT* p.R406W (insular cortex) and control (parietal cortex) brains and the –log_10_ *P*-value for each gene. Black nodes: genes with FDR B-Y < 0.05. **d** Heat map of the 61 genes differentially expressed in brains from *MAPT* p.R406W carriers compared to controls. **e** Venn diagram of differentially expressed genes between *MAPT* p.R406W and controls. **f** Correlation of the effect size (shown as log2 fold change) of the genes differentially expressed between *MAPT* p.R406W vs. isogenic control neurons and *MAPT* p.R406W (insular cortex) vs. control (insular cortex) brains. **g** Correlation of the effect size of the genes differentially expressed between *MAPT* p.R406W vs. isogenic control neurons and *MAPT* p.R406W (insular cortex) vs. control (parietal cortex) brains. **h** Relatedness of the 61 differentially expressed genes replicated in iPSC-derived neurons and human brains. The sizes of the gene nodes are proportional to GeneMANIA scores of the genes (the degrees to which the genes are related). Black node: *MAPT* gene; blue nodes: genes directly interacted with *MAPT*; Yellow nodes: drug target genes; green nodes: genes encompassed by FTD risk variants; red node: drug target gene encompassed by FTD risk variants. **i** GO term enrichment. **j** KEGG pathway enrichment

Next, to define the differentially expressed genes that lead to the major changes in the transcriptome analyses, we applied the strict B-Y FDR on the 18,537 genes that were measured across all the cells. We found that 328 genes are differentially expressed between neurons expressing *MAPT* p.R406W and isogenic controls (Fig. 2d and Fig. 3a; Supplemental Table 3).

### Gene expression patterns in brain tissue from *MAPT* p.R406W carriers overlaps with those observed in human stem cell models

Given that FTLD with *MAPT* p.R406W is a neurodegenerative disease and stem cell systems are thought to represent relatively immature states, we were uncertain as to whether iPSC-derived neurons could capture molecular phenotypes relevant to a disease that develops clinical symptoms several decades after birth or whether the differentially expressed genes only capture different states of development. Combining the differential expression analyses from iPSC-derived neurons and brains should allow us to identify genes altered as a function of the *MAPT* mutation and involved in pathology rather than those genes implicated in neurodevelopment (iPSC-only artifacts) or in general neurodegeneration and cell death-related processed (autopsied brain-only artifacts). To determine whether any of the 328 genes differentially expressed between *MAPT* p.R406W and isogenic controls are related to disease pathogenesis, we examined brain tissue from cases of FTLD with *MAPT* p.R406W and non-mutation carriers free of pathology (Fig. 3). We performed RNA-seq in the insular cortex, a region affected early in disease^46–48^. Due to the limited amount of tissue available from *MAPT* p.R406W carriers, we expanded our dataset by including additional control brain samples (parietal cortex) from non-carriers free of pathology (Fig. 3).

Principal component analysis of the whole transcriptome illustrates that brains from FTLD with *MAPT* p.R406W also cluster separately from non-mutation carriers free of pathology (Supplemental Fig. 2). We also demonstrate that control brains from the insular and parietal cortices cluster similarly, suggesting there are few overall differences in gene expression between these two brain regions and analyses including both regions should not bias our results (Supplemental Fig. 2). Of the 328 genes differentially expressed between *MAPT* p.R406W and isogenic control neurons, we found that 61 of these genes were also differentially expressed in the same direction with a B-Y FDR < 0.05 between the insular cortex of FTLD with *MAPT* p.R406W and controls and between the insular cortex and partial lobe of mutation carriers and controls (Fig. 3; Supplemental Table 4).

We found that these 61 differentially expressed genes exhibited similar effect sizes in the neurons and brains (Fig. 3f, g; Pearson correlation *R* = 0.969 and *P* = 1.645 × 10^−37^ for neurons vs. parietal cortices and Pearson correlation *R* = 0.968 and *P* = 6.302 × 10^−37^ for neurons vs. insular cortices). Interestingly, only three genes were differentially expressed but in the opposite direction in the neurons and brains (Supplemental Tables 3 and 4). Importantly, this suggests that our iPSC-derived neuronal system is capable of capturing gene expression changes similar to those present in human brains at autopsy. Thus, these 61 differentially genes represent genes that are altered by the *MAPT* p.R406W mutation and are likely to be relevant to disease pathogenesis.

### Genes dysregulated by *MAPT* p.R406W are enriched for pathways involving GABA receptors and pre-synaptic function

We next sought to determine whether the 61 genes differentially expressed between *MAPT* p.R406W carriers and controls in iPSC-derived neurons and human brains were enriched in any specific pathways or whether they interacted with one another. GeneMANIA analysis revealed that more than half of the 61 genes directly interacted with *MAPT* (Fig. 3h). The GeneMANIA interactions of the 61 genes span over multiple relationships: co-expression (70.52%), co-localization (16.07%), physical interaction (7.89%), co-pathway (2.9%), computationally predicted (2.26%), genetic interaction (0.33%) and shared protein domain (0.03%). Importantly, the GABA-associated genes *SNAP25* and *SYT1* are hubs in the interaction network (Supplemental Table 5). Hub genes are defined by the total number of nodes directly connected to a given node; rankings of hub genes also factor in betweenness and closeness centrality. Reconstructing the GeneMANIA network without co-expression revealed that the top two types of gene relationships are co-localization (55.28%) and physical interaction (26.22%). Twenty-six genes were directly connected to *MAPT*, and GABA-associated genes *SYT1* and *SNAP25* remained the hub genes of the network (Supplemental Fig. 3).

Next, we determined if these 61 genes were enriched in specific biological pathways. We found that the 61 genes are enriched for functions related to calcium-dependent presynaptic function (FDR-adjusted *P* = 2.2 × 10^−2^; *STMN2*, *SNAP25*, *SYT1*, and *CALM1*), kinesin binding (FDR-adjusted *P* = 4.85 × 10^−2^; *KIFAP3*, *SNCA*, *CROCC*; Fig. 3i) and pathways involving GABAergic signaling (FDR-adjusted *P* = 5.43 × 10^−3^; *GABRB2*, *GABRG2*, *NSF*, *NGN2*, *PRKACB*; Fig. 3j). Importantly, these enriched pathways appear to be functionally related, as *SNAP25* is calcium-dependent protein binding that is critical for evoked GABA release and is expressed in the presynaptic terminals of mature GABAergic neurons^49^.

In addition to evaluating gene ontologies and biological pathways that are enriched by the 61 differentially expressed genes, we tested whether these genes are enriched for known drugs using Webgestalt. These analyses identified 32 GABA agonists and antagonists that target *GABRB2* and *GABRG2* (Supplemental Table 6). This served as an important proof of concept that we can leverage our molecular and biological findings with publicly available data on drug interactions to identify and prioritize putative FDA-approved drug targets and repurpose these molecules as potential treatments for FTLD with *MAPT* p.R406W.

### GABA receptors

**Fig. 4:**
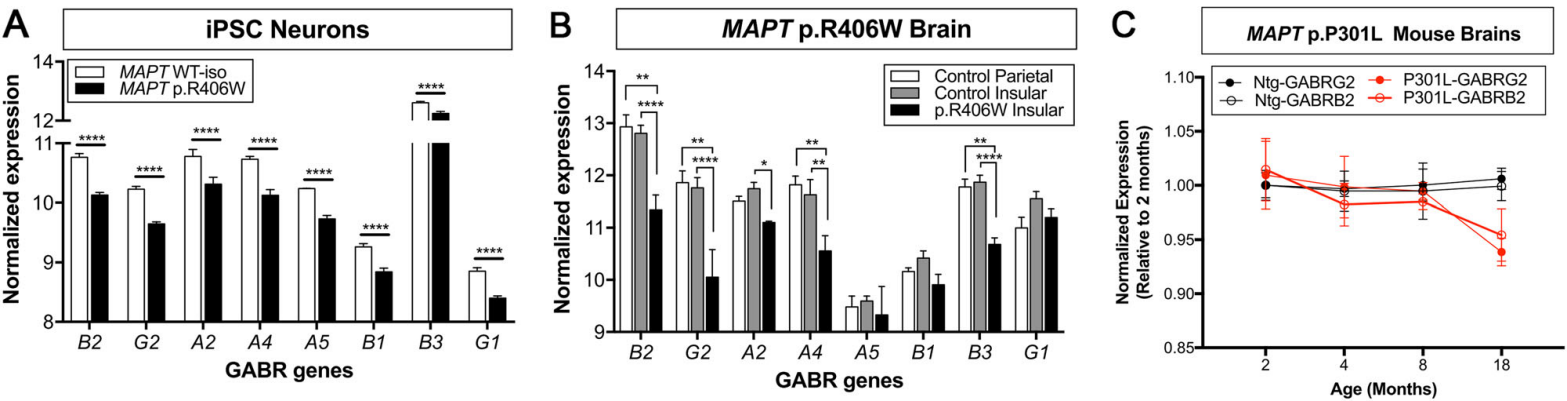
GABA receptor gene expression is reduced in primary tauopathies. GABA receptor gene expression in: **a** iPSC-derived cortical neurons carrying *MAPT* p.R406W (black bars) and isogenic controls (WT-iso; white bars). **b** Brains from *MAPT* p.R406W carriers (insular cortex, black bars) and controls (insular cortex, gray bars; parietal cortex, white bars). **c**. Hippocampus of *MAPT* p.P301L mouse (red lines) and non-transgenic controls (black lines). Graph represents mean ± SEM. *P < 0.05; **P < 0.01; ***P < 0.001; ****P < 0.0001

Given the enrichment in GABAergic signaling among the 61 genes differentially expressed between *MAPT* p.R406W and controls in iPSC-derived neurons and human brains, we examined expression of all genes encoding GABA receptor subunits (Fig. 4; Supplemental Table 7). In iPSC-derived neurons expressing *MAPT* p.R406W, we observed broad downregulation of GABA receptor genes: *GABRB2*, *GABRG2*, *GABRA2*, *GABRA4*, *GABRA5*, *GABRB1*, *GABRB3*, *GABRG1* (Fig. 4a). Similarly, most of these GABA receptor genes were also downregulated in human brains from *MAPT* p.R406W carriers, with the exception of *GABRA5, GABRB1*, *GABRG1* (Fig. 4b), although some of these genes did not pass the stringent multiple correction we applied in the initial analyses. It is important to note that the GABA_B_ receptors form functional heterodimers composed of GABA B1 and GABA B2 subunits. The GABA B2 subunit, which is encoded by *GABRB2*, is essential for the functional expression of the receptor dimer at the cell surface. Interestingly, the observed change in receptor expression was specific, as genes encoding subunits for AMPA and NMDA receptors were not differentially expressed (Supplemental Table 8).

### Mouse models of tauopathy display altered GABA receptor expression profiles

Our stem cell models and human brain tissue samples capture a single time point in the progression of the disease; thus, we next sought to resolve whether GABA receptor gene expression is impaired early in disease or as a consequence of pathologic events. To begin to address this question, we examined temporal expression of *Gabbr1*, *Gabrb2*, *Gabrb3*, and *Gabrg2* in a mouse model of tauopathy that overexpresses human *MAPT* p.P301L^37^. We observed robust and consistent down regulation of *Gabbr1*, *Gabrb2*, *Gabrb3*, and *Gabrg2* at 18 months of age in *MAPT* p.P301L mice compared with non-transgenic controls but not prior (Fig. 4c; Supplemental Fig. 4). This time point coincides with the presence of overt tau aggregation and neurodegeneration in this model^37^. Thus, we hypothesize that *MAPT* mutations, including p.P301L and p.R406W are sufficient to induce pathologic changes that lead to altered GABAergic signaling.

### Common variants in GABA receptor genes may influence FTD risk

To determine whether the 61 differentially expressed genes are involved more broadly in FTD, we asked whether variants present in the 61 differentially expressed genes confer risk for FTD. We used summary statistics from the largest GWAS to date of FTD (*n* = 6462)^39^. We extracted all SNPs that occur within 5 kb of the 61 differentially expressed genes. Five genes, including the GABA receptor gene, *GABRB2*, were found to be marginally associated with FTD risk (Table 2; Supplemental Table 9).

**Table 2:** Enrichment of genes differentially expressed by *MAPT* p.R406W among FTD risk SNPs

SNP	Chromosome	Allele 1	Allele 2	β	*P* value	Nearest gene	Location in Gene
rs9531160	13	T	C	−0.2723	3.54E-04	*DCLK1*	Intron
rs6743717	2	A	G	0.1804	3.63E-04	*FAM49A*	Intron
rs17521304	5	T	C	0.225	5.64E-04	*GABRB2*	Intron
rs199533	17	A	G	−0.1948	5.94E-05	*NSF*	Exon; K720K
rs3738684	1	A	T	0.2893	8.49E-05	*OBSCN*	Intron

**Fig. 5:**
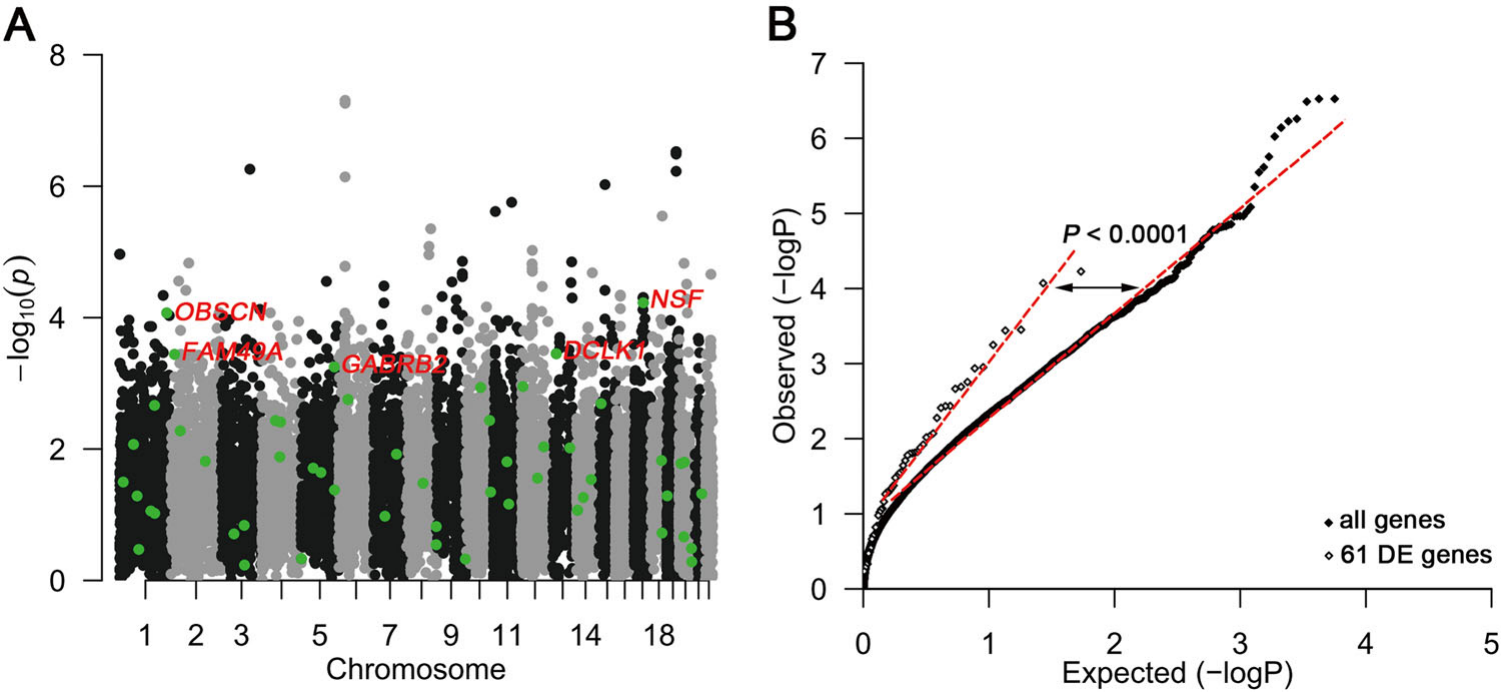
Enrichment of *MAPT* p.R406W dysregulated genes in genetic risk for FTD. To determine whether the 61 verified differentially expressed genes in *MAPT* p.R406W carriers were enriched for FTD risk, *i*-GSEA4GWAS was employed for enrichment analysis of FTD risk. **a** Gene-based Manhattan plot of the enrichment analysis of the genetic association signals from FTD summary statistics for the 61 verified differentially expressed genes. **b** Comparative QQ plot of the genetic association signals from FTD summary statistics between all genes and the 61 verified differentially expressed genes

To determine whether the 61 differentially expressed genes were significantly enriched for FTD risk variants, we performed *i*-GSEA4GWAS. The 61 differentially expressed genes were defined as a gene set and enrichment analysis was performed to determine whether the FTD risk variants were significantly enriched within the gene set. The 61 genes were significantly enriched for FTD risk variants (Fig. 5a; *P*-value < 0.0001). Furthermore, we demonstrate that the SNPs within the 61 differentially expressed genes are more significantly associated with FTD risk than expected by chance (Fig. 5b). To demonstrate specificity of this genetic enrichment, we sought to determine whether genes differentially expressed in AD, SCZ, BP, and ASD (Supplemental Table 10) were also significantly enriched among FTD risk variants^42^. We observed no significant enrichment (Supplemental Fig. 5). These analyses provide further evidence to support an important role for these 61 genes in FTD.

### GABA receptor involvement in other neurodegenerative diseases

**Fig. 6:**
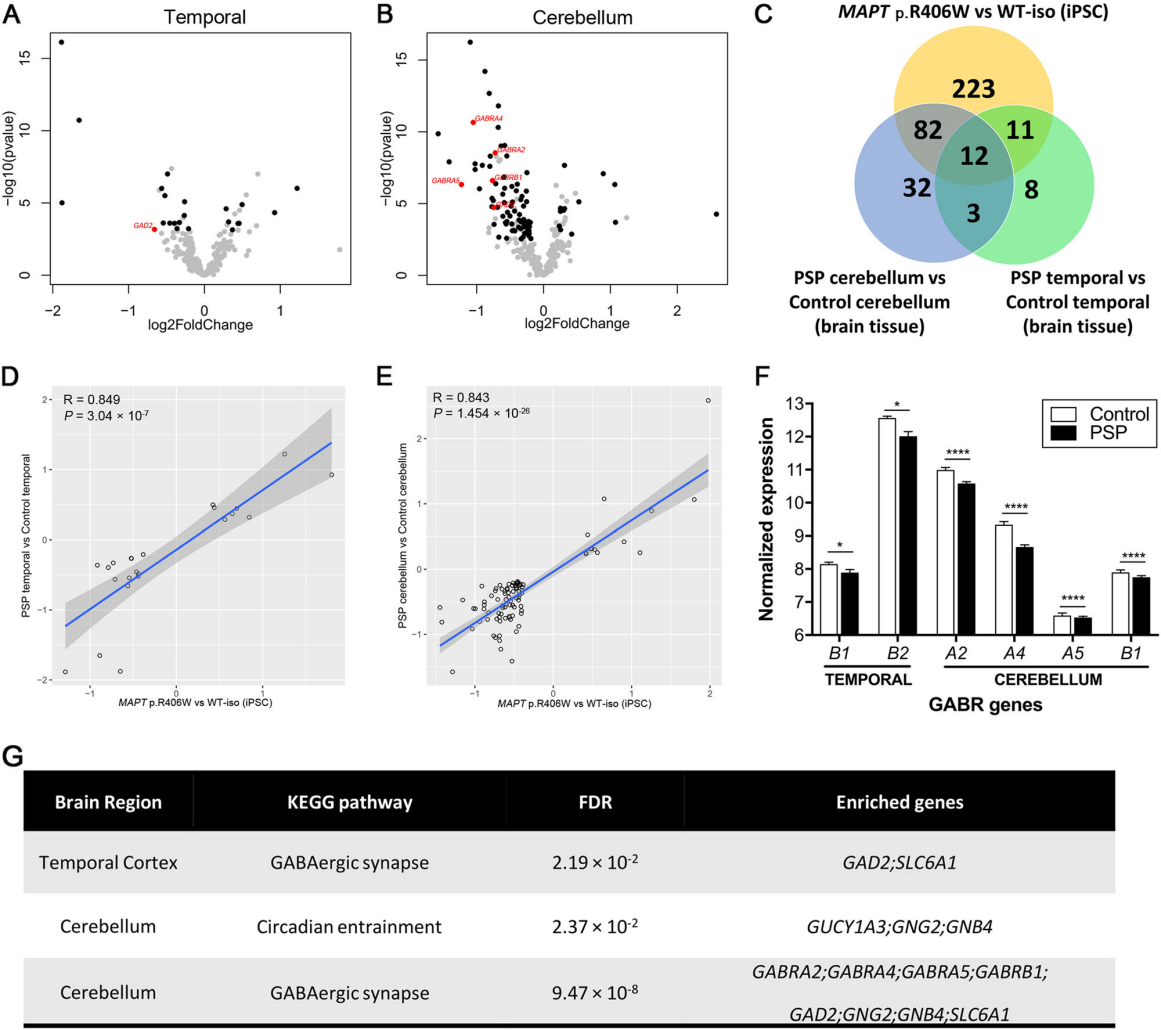
Genes differentially expressed by *MAPT* p.R406W are also altered in PSP brains. To determine whether the 328 differentially expressed genes in iPSC-derived neuronal models capture changes in gene expression that are relevant to primary tauopathy, we tested whether these 328 genes were differentially expressed in human brains from PSP patients. **a** Volcano plot of the 328 genes showing log2 fold change between PSP and control brains (temporal cortex) and the –log_10_ *P*-value for each gene. Black nodes: genes with FDR B-Y < 0.05. **b** Volcano plot of the 328 genes showing log2 fold change between PSP and control brains (cerebellum) and the –log_10_ *P*-value for each gene. Black nodes: genes with FDR B-Y < 0.05. **c** Venn diagram of differentially expressed genes between PSP and control brains. **d** Correlation of the effect size of the genes differentially expressed between *MAPT* p.R406W vs. isogenic control neurons and PSP vs. control brains (temporal cortex). **e** Correlation of the effect size of the genes differentially expressed between *MAPT* p.R406W vs. isogenic control neurons and PSP vs. control brains (cerebellum). **f** Reduced GABA receptor genes in PSP brains. Graph represents mean ± SEM. **g** KEGG pathway enrichment

There is pathologic overlap between FTLD with *MAPT* p.R406W and other primary tauopathies, including PSP and CBD^50^. Thus, we next sought to determine whether the 328 genes differentially expressed in in iPSC-derived neurons from *MAPT* p.R406W carriers and isogenic controls capture aspects of disease pathogenesis that are more broadly related to another primary tauopathy, PSP. We performed differential expression analyses in two brain regions affected in disease, temporal neocortex and cerebellum, from PSP cases and pathology-free controls. We found that 23 genes were differentially expressed in the same direction in the stem cell model and in the temporal cortex of PSP cases compared to controls (Fig. 6; Supplemental Table 11). We also found that 94 genes were differentially expressed in the same direction in the stem cell model and in the cerebellum of PSP cases compared to controls (Fig. 6b, c; Supplemental Table 11). The effect size of the expression of these genes were highly correlated in temporal cortex (Fig. 6d; Pearson correlation *R* = 0.849 and *P* = 3.04 × 10^−7^) and cerebellum (Fig. 6e; Pearson correlation *R* = 0.843 and *P* = 1.454 × 10^−26^). In total, 12 of the 328 genes (*NALCN, CX3CR1, DSP, MGP, CELSR1, KIAA1107, EMB, GREB1L, GAD2, LGI2, SLC6A1*, and *TONSL*) were differentially expressed in the same direction in the stem cell models and both PSP brain regions (Fig. 6c). Only a single gene, *KIAA1107*, was significantly reduced in stem cell models and brains from *MAPT* p.R406W carriers and PSP brains (Supplemental Fig. 6C; Supplemental Table 11). *KIAA1107* is an uncharacterized protein that has been implicated in white matter lesions in multiple sclerosis and has been implicated in risk for FTD, PSP, and CBD^39,51–53^.

To determine whether genes involved in GABAergic signaling are similarly differentially expressed in PSP brains, we performed pathway and differential expression analysis. We found that the GABAergic synapse pathway was significantly enriched among the differentially expressed genes in the temporal cortex and cerebellum of PSP cases (Fig. 6g). We also found that *GABRB1* and *GABRB2* were significantly lower in the temporal cortex of PSP brains and that *GABRA2*, *GABRA4*, *GABRA5*, and *GABRB1* were significantly lower in the cerebellum of PSP brains compared with pathology-free controls (Fig. 6f). Of note, *GAD2*, which was among the 12 differentially expressed genes in both PSP brain regions and in the stem cell model, encodes an enzyme that catalyzes the decarboxylation of glutamate to GABA and CO_2_.

To determine whether the 328 differentially expressed genes in the stem cell model of *MAPT* p.R406W represent genes that are broadly related to neurodegenerative disease, we examined RNA-seq data from the brains of autosomal dominant AD carrying *PSEN1* mutations and from brains with FTLD-TDP that are sporadic in nature. We failed to observe any of the 328 differentially expressed genes in either dataset (Supplemental Fig. 6A-B; Supplemental Table 12). Additionally, we found that the 328 genes are not significantly differentially expressed in the human developing brain (Supplemental Fig. 7). Thus, these 328 genes do not capture disease processes related to AD, where tauopathy is an event secondary to β-amyloidosis; disease processes related to FTLD associated with TDP-43 inclusions; nor developmental processes. Furthermore, these results indicate that the genes shared between the stem cell model and human brains from *MAPT* p.R406W carriers did not occur by chance.

## Discussion

Here, we use a systems biology approach to identify molecular drivers of FTLD with *MAPT* p.R406W. By leveraging transcriptomics data from human stem cell models and human brain tissue and bioinformatic approaches, we demonstrate that genes enriched in presynaptic function are altered in FTLD with *MAPT* p.R406W. Furthermore, we demonstrate that GABA receptor expression is altered as a function of disease.

The goal of using patient-derived cell culture models is to develop tractable, human platforms that recapitulate disease-specific and patient-specific phenotypes. However, reagent costs and experimental variables preclude us and the scientific community in general from studying exceedingly large numbers of patient lines in parallel. Here, we demonstrate that even in a relative small number of patient stem cell lines and paired human brain tissues have sufficient power to detect molecular drivers of disease.

In this study, we demonstrate that GABA receptor genes are reduced in FTLD with *MAPT* p.R406W. GABA receptors occur in the pre-synaptic and post-synaptic regions where they function to bind GABA, the major inhibitory neurotransmitter in the brain. During development, GABA is the major excitatory neurotransmitter in the brain prior to the maturation of glutamatergic synapses, where it plays critical roles in neural progenitor cell proliferation, elongation of neurites and formation of synapses. There are two major classes of GABA receptors: GABA_A_ and GABA_B_. GABA_A_ receptors compose the ligand-gated ion channel complex, while GABA_B_ receptors are G protein-coupled neuromodulatory receptors. While we observed a marginal effect of *MAPT* p.R406W on GABA_A_ receptor subunit genes (Supplemental Table 2 and 3), in the iPSC-derived neurons, human brains and mouse model, GABA_B_ receptors were significantly reduced in *MAPT* mutation carriers. Importantly, *GABRB2* was robustly reduced across the model systems. *GABRB2* encodes the GABA B2 subunit, which is the major regulator of the functional expression of the receptor dimer on the cell surface^54^. Thus, reduction of this gene could have a significant effect on the number of functional GABA receptors at the synapse. Beyond the eight GABA receptor genes that were identified in the stem cell model, we also observed differential expression of genes related to GABA signaling. *SNAP25* is calcium-dependent protein binding that is critical for evoked GABA release and is expressed in the presynaptic terminals of mature GABAergic neurons^49^. *GAD2* encodes an enzyme that catalyzes the decarboxylation of glutamate to GABA and CO_2_^55^. Interestingly, *SYT1*, an integral membrane protein involved in synaptic vesicles, co-localizes with *KIAA1107*, the gene differentially expressed in stem cell models and brains from *MAPT* p.R406W carriers and PSP brains, in neurons^56^.

Defects in GABAergic signaling have been previously implicated in tauopathies. A mouse model of tauopathy expressing *MAPT* p.P301L exhibited a reduction in hippocampal GABAergic interneurons^57^. Enhanced late-phase long-term potentiation was observed in these mice that could be rescued with zolpidem, a GABA_A_R agonist^57^. Several prior studies have reported qualitative evidence for the role of GABAergic neurons in FTLD-tau*. Caenorhabditis elegans* and stem cell models of an FTLD-tau risk variant in *MAPT* (*MAPT* p.A152T) exhibit abnormal morphology and degeneration of GABA-positive cells^58,59^. GABA_A_ receptor activity may regulate tau phosphorylation^60^. GABAergic interneurons are reduced in some FTLD-tau brains, including PSP^61,62^. PSP brains also exhibit GABA_A_ receptor loss by flumazenil-PET^63,64^. However, how these defects fit within molecular cascade that leads to disease remains unresolved. Importantly, these studies did not reveal whether changes in GABAergic neurons and GABA receptors were a consequence of early molecular dysfunction or end-stage neurodegeneration. Our findings suggest that *MAPT* p.R406W is necessary and sufficient to induce changes in GABA receptor genes and other genes that regulate GABA function. Furthermore, we demonstrate that this is maintained in human tissue, suggesting that the event triggered by the *MAPT* mutation is maintained throughout the disease course.

Drug enrichment analysis revealed a number of GABA receptor agonists and antagonists that act on the genes differentially expressed in our stem cell model and human brains. The clinical syndrome underlying FTLD-tau pathology in the brain presents with behavioral abnormalities, language and executive dysfunction, memory loss, and other changes^65,66^. GABA receptor agonists have been considered as a possible treatment for these clinical phenotypes^67^. However, our findings suggest that drugs that increase GABA receptor expression or enhance their function could be a therapeutic strategy that targets central mechanism of disease.

iGSEA4GWAS analysis showed that the 61 verified differentially expressed genes in *MAPT* p.R406W carriers are generally enriched with more FTD risk variants than average genes, indicating a genetic basis for the perturbed expression profile. Interestingly, we found that GABA-associated genes, *GABRB2* and *NSF*, are within susceptibility loci for FTLD. Two marginal risk variants (*P*-value = 7.24 × 10^−3^ for rs13178594 and *P*-value = 9.14 × 10^−3^ for rs194072) were also mapped to *GABRB2* in the GWAS for PSP risk^68^. Of note, the haplotype block constituted by genetic susceptibility loci of FTLD mapped to *NSF*, or *MAPT* H1 haplotype^69^, is a significant GWAS hit shared with FTD, PSP, CBD, and Parkinson disease^53,68,70^.

Our results also indicate that the iPSC model of *MAPT* p.R406W has some correlation with PSP, implicating the ability of the iPSC model of *MAPT* p.R406W to capture disease mechanisms related to other primary tauopathies. The reduced GABA receptor genes, GABAergic pathway, and drugs targeting GABA receptors were also identified in PSP, suggesting the loss-of-function of GABAergic neurons is a common mechanism of tauopathy^57,71,72^. However, the iPSC model of *MAPT* p.R406W is uninformative with regards to *PSEN1* mutations in AD or FTLD-TDP, further supporting its specificity for primary tauopathy.

Performing transcriptome analyses (differential expression and network analyses) in human brain tissue has been highly informative in identifying potential co-expression networks. Constructing gene regulatory networks from 1647 post-mortem brain tissues of AD and pathology-free controls revealed dysregulation of genes involved in immune and microglia networks and identified *TYROBP* as a key regulator of these networks^73^. More recently, multiscale networks in human AD brains suggest that human herpesvirus subtypes may impact AD risk via regulation of genes involved in APP processing^74^. However, it remains unclear whether these networks are implicated in disease pathogenesis or an unrelated event in the neurodegenerative process. At the same time, performing similar analyses in iPSC-derived neural cells presents huge potential, but it is not clear whether this cellular system recapitulates the events that lead to disease late in life. The results of this study indicate that iPSC-derived neurons are highly informative and recapitulate the pathogenic events leading to disease. While, of course, not all the transcriptome changes in these models will be important to disease, when coupled with human brain tissue, it is clear that the differentially expressed genes shared between the two models are implicated in disease and are unlikely to be a mere artifact. These results also confirm that using human brain tissue from deceased individuals can be informative, but when paired with additional models, our data indicate that it may not be necessary to study hundreds of samples to identify reliable and replicable signals. In this study, we paired the human brain tissue with iPSC-derived neurons because the *MAPT* gene is mainly expressed in neurons, but we recognize that we may be missing other relevant pathways involved in non-neuronal cell types, including oligodendrocytes, astrocytes, and microglia cells. Thus, future studies using iPSC-derived glia can lead to the identification of additional pathways implicated in disease. This study indicates that pairing iPSC-derived neurons with human tissue is a highly powerful and informative approach that helps to identify novel pathways involved in disease. We predict that similar study designs may be helpful in identifying pathogenic pathways leading to neurodegeneration in other diseases and in other genes. This is especially important for studies trying to characterize different events that lead to disease in rare (Mendelian mutations causing AD, PD or FTD) or low frequency variants (i.e., *TREM2* p.R47H) with a large effect size conditions, where it is difficult to identify a large number of brain samples that carry these variants.

## Electronic supplementary material

Supplemental Figures 1-7

Supplemental Tables 1-12

## Data Availability

The datasets analyzed during the current study are available from these sources: Mayo Clinic Brain Bank: https://www.synapse.org/#!Synapse:syn5550404; Mount Sinai Brain Bank: https://www.synapse.org/#!Synapse:syn3157743; The Knight-ADRC: https://www.synapse.org/#!Synapse:syn12181323. According to the data request terms, DIAN data are available upon request: http://dian.wustl.edu.
